# A Novel Link-to-System Mapping Technique Based on Machine Learning for 5G/IoT Wireless Networks

**DOI:** 10.3390/s19051196

**Published:** 2019-03-08

**Authors:** Eunmi Chu, Janghyuk Yoon, Bang Chul Jung

**Affiliations:** Department of Electronics Engineering, Chungnam National University, Daejeon 34134, Korea; emchu@cnu.ac.kr (E.C.); 201501758@o.cnu.ac.kr (J.Y.)

**Keywords:** link-to-system mapping, exponential effective SNR mapping (EESM), physical-layer abstraction, system-level simulation, machine learning, deep neural network (DNN)

## Abstract

In this paper, we propose a novel machine learning (ML) based link-to-system (L2S) mapping technique for inter-connecting a link-level simulator (LLS) and a system-level simulator (SLS). For validating the proposed technique, we utilized 5G K-Simulator, which was developed through a collaborative research project in Republic of Korea and includes LLS, SLS, and network-level simulator (NS). We first describe a general procedure of the L2S mapping methodology for 5G new radio (NR) systems, and then, we explain the proposed ML-based exponential effective signal-to-noise ratio (SNR) mapping (EESM) method with a deep neural network (DNN) regression algorithm. We compared the proposed ML-based EESM method with the conventional L2S mapping method. Through extensive simulation results, we show that the proposed ML-based L2S mapping technique yielded better prediction accuracy in regards to block error rate (BLER) while reducing the processing time.

## 1. Introduction

With technological advance of 4G LTE/LTE-A (Long Term Evolution-Advanced) cellular communications, the number of wireless smart devices has increased explosively. Wireless smart devices have being applied in various services such as IoT (Internet of Things) communications, autonomous vehicles communication, augmented reality service, etc. [1]. In the near future, a wide range of service requirements will be demanded due to advent of many use cases [2]. However, 4G LTE/LTE-A systems have several limitations to satisfy requirements of various services.

As the next version of 4G LTE/LTE-A, 3GPP Release 15 (Rel-15) has specified 5G new radio access (NR) and core technologies from December 2017 and it is known as 5G NR. 5G NR access technology is evolving to support a variety of service requirements and it contains enhanced mobile broadband (eMBB), massive machine type communications (mMTC), and ultra reliable low latency communications services (URLLC) as 5G representative services [3]. To support low latency service, 5G NR system has embraced new flexible frame structures different from 4G LTE/LTE-A system with a fixed sub-carrier interval. Moreover, to support high data rate and high traffic service for eMBB use cases, 5G NR systems are expanding to high frequency bands above 6 GHz. Accordingly, 5G NR system will be mainly operated on high frequency band with a wide bandwidth [4].

In a wide band channel, a transport block (TB) is allocated into *N* narrow band channels and each narrow band channel goes through a different fading condition on its own sub-carrier. Therefore, user equipment (UE) experiences different post-processing signal to interference plus noise ratio (SINR) over every sub-carrier. In a traditional narrow band channel, block error rate (BLER) is estimated from a curve of mean SINR and mean BER. On the contrary, in the wide band channel, different *N* post-processing SINRs are mapped to the averaged post-processing SINR. Since the concept of the averaged post-processing SINR is defined as an effective SNR, this many-to-one mapping is called an effective SNR mapping (ESM) technique. Besides, ESM technique is used for the purpose of physical layer abstraction when evaluating a system-level simulator (SLS). A simplified link-level simulator (LLS) helps SLS reduce complexity of computation and it can help improve a simulator performance. Since the concept of a physical level abstraction for SLS is reflected, this is also called a link-to-system (L2S) mapping technique. Accordingly, a L2S mapping is that post-processing SINRs extracted from LLS are mapped to an effective SNR and BLER is predicted by the effective SNR, thus, it is called effective SNR mapping (ESM).

In prior studies, many researchers analyzed exponential effective SINR mapping (EESM) [5,6] and mutual information based ESM [7] as representative L2S mappings. In [8], effective SNR is analyzed on the side of uplink. In [9], impact of L2S is analyzed on the side of system level. However, there are too many data extracted from LLS as well as too much processing time is need to find EESM mapping parameters for various cases. Moreover, loss incurs due to an inaccuracy from additive white Gaussian noise (AWGN) curve of SNR and BLER.

Therefore, recently, researchers studied ML-based link abstraction models. In [10], support vector machine is used to enable ML classification for fast adaptive modulation coding. This scheme exploits measurement of single TB success or failure to train the classifier. In [11], a ML method based on a logistic regression is proposed. To predict a TB success or failure, their basic model uses mean and standard deviation of the SINR set, modulation rate, and TB size as input variables. To improve the estimation accuracy, adding terms of higher order or combinations of input variables are used in an enhanced model.

To utilize ML-based link abstraction models that have been studied thus far, ML algorithms should be applied on both of an evolved Node-B (eNB) and UE sides. However, since the number of UEs is too large, it is difficult to embed ML algorithms in all UEs. Some UEs can directly apply ML algorithms while other UEs should take the existing EESM method. Therefore, eNBs still need the existing EESM method. In our previous work [12], we proposed a ML-based EESM method where training data are learned by deep neural network (DNN) regression and L2S mapping based on EESM is executed by optimization algorithms under 4G LTE system environments. We showed that the processing time and accuracy are improved. However, L2S mapping-related previous studies including our previous work have been done based on 3G/4G cellular networks. Our previous work is meaningful as the existing results under SISO condition of 4G LTE systems are applied to machine learning techniques.

Recently, the standards of 5G NR systems based on Rel-15 decided that LDPC channel coding on data plane is preferred and clustered delay line (CDL) and tapped delay line (TDL) as 5G channel models are used. Since the standards of 5G NR based on Rel-15 are continually progressing, the performance results for AWGN channel of simple model are not easy to obtain. To develop LLS, SLS, and network-level simulator (NS) for 5G NR based on Rel-15, a Korean collaborative research project developed 5G K-Simulator during August 2016–February 2019 [13,14]. Our role was to develop a L2S mapping method as an inter-connection between the LLS and the SLS in 5G K-Simulator. To the best of our knowledge, were were the first to design a machine learning based L2S mapping under 5G NR systems based on Rel-15. The main contributions of this paper are as follows:

(1) AWGN curves based on 5G NR system are provided. In conventional L2S mapping schemes, an AWGN curve is required to estimate block error rate (BLER) on SLS. We provide the AWGN curve of SNR-BLER under 5G NR systems and compare the AWGN curves of SNR-BLER between 4G LTE system and 5G NR systems.
(2) The values of SNR satisfying at a target BLER = 0.1 (10%) are provided through AWGN curve. 3GPP 4G LTE and 5G NR-standards are specified to set the target BLER of 0.1, i.e., 10%. In SLS, signal-to noise ratio (SNR) can be measured immediately, but the calculation of BLER is burden because CRC decoding has to be performed. When a measured BLER is lower than the target BLER, UE can change to the index of higher CQI to support high data rate. Therefore, we provide the values of SNR thresholds under an unknown BLER satisfying the target BLER of 0.1 through the simulation results.
(3) The methodology for 5G NR L2S mapping is provided based on ML. The optimal parameters for L2S mapping depend on a given environment. In addition, 5G NR systems have flexible frame structures. Therefore, we provide the methodology for 5G NR L2S mapping based on ML to extract the data for any cases and find the optimal mapping parameters.
(4) The optimal mapping parameters for L2S mapping are provided in SISO and 2 × 2 MIMO cases under given conditions.

This paper is organized as follows. In Section 2, we introduce 5G NR system and main difference of 4G and 5G systems. In Section 3, we describe the methodology for 5G NR L2S mapping. In Section 4, we propose machine learning-based effective SNR mapping procedure. In Section 5, we validate the proposed scheme and show the simulation results of the proposed scheme. Finally, we draw conclusions in Section 6. The abbreviations commonly used in this paper are summarized as Table 1.

## 2. Background on 5G NR

3GPP Rel-15 was approved for NR standards in December 2017, referred to as 5G NR. Different from 4G LTE-A systems with fixed sub-carrier intervals, 5G NR systems have newly flexible frame structures to provide various services. Various transmission frame settings are available by introducing sub-carrier interval parameter μ called numerology. 5G NR systems can set μ from 0 to 5, and the value of μ determines the sub-carrier interval. As the numerology value μ increases by one, the sub-carrier interval is doubled. Compared to 5G NR systems, 4G LTE/LTE-A systems only use a fixed sub-carrier interval of 15 kHz (μ=0), therefore, it can accommodate various services.

The most innovative change of 5G NR system is channel coding. Turbo codes [15], low-density parity-check (LDPC) code [16], and polar code [17] were designated as candidate channel codings. In particular, LDPC code and the polar code have been actively studied, and the performance of LDPC code has been shown to be close to the Shannon limit. In a recent study, LDPC code enables fast encoding/decoding with high error correction capability, and achieves high speed, low delay, low cost, and high reliability for 5G communication services. At the first-phase of Rel-15, LDPC code replaces the Turbo code used in 4G LTE data channels, while Polar code replaces the Tail Biting Convolutional Codes used in 4G LTE control channels.

3GPP standards technical reports of TR 38.900 [18] and TR 38.901 [19] define clustered delay line (CDL) and tapped delay line (TDL) as 5G channel models for LLS. Each channel model consists of three non-line-of-sight channels of A, B, and C types and two line-of-sight channels of D and E types. These channel models support bandwidth of up to 2 GHz in the 500 MHz to 100 GHz operating frequency.

The CDL model [18,19] is designed as a signal arriving at the receiver antenna dispersed into 20 signals as it passes through each cluster in the channel. At this time, the delay, power, and four kinds of angles (azimuth angles of arrival and departure, and zenith angles of arrival and departure) are defined. In the case of the TDL model, instead of defining each cluster parameter in the channel as in the CDL model, only the power delay profile of each taps is defined for the entire channel. Therefore, it is a simplified form in which the four angular values do not appear, and the process of distributing signals passing through each cluster to 20 signals is not modeled. It is also possible to generate TDL models by assuming non-isotropic antennas such as directive horn antennas or array antennas. The TDLs and the spatial-filtered TDLs can be used with the correlation matrices for MIMO link-level simulations.

In contrast, 4G LTE-A system models the wideband characteristics of the channel as a TDL. Each tap independently experiences a fading characteristic by an azimuth direction of departure and direction of arrival angular spectrum. Since the mean direction and angular spread are fixed, TDL represents stationary channel conditions in 4G LTE-A system.

## 3. Methodology for 5G NR Link-to-System Mapping

5G K-Simulator [13] is a Korean collaborative research project consisting of several Korean universities and companies to develop a 5G NR simulator. At the start of the project, there was no widely accepted L2S mapping suitable for 5G NR system. Our role was to develop a L2S mapping method as an inter-connection between the LLS and the SLS in 5G K-Simulator. In this section, we deal with considerations for the 5G NR system in Section 3.1, and we describe the preliminary phase to be performed in the LLS simulation for L2S mapping in Section 3.2. Finally, we explain a L2S mapping method for 5G NR systems in Section 3.3.

### 3.1. System Configuration/Setup

5G NR systems have been studied in many test scenarios in 3GPP TR 38.802 [20]. However, this paper focuses on parameter settings for L2S mapping test among many scenarios. Table 2 summarizes the system parameters used in this paper, and details are described below.

**Figure 1:**
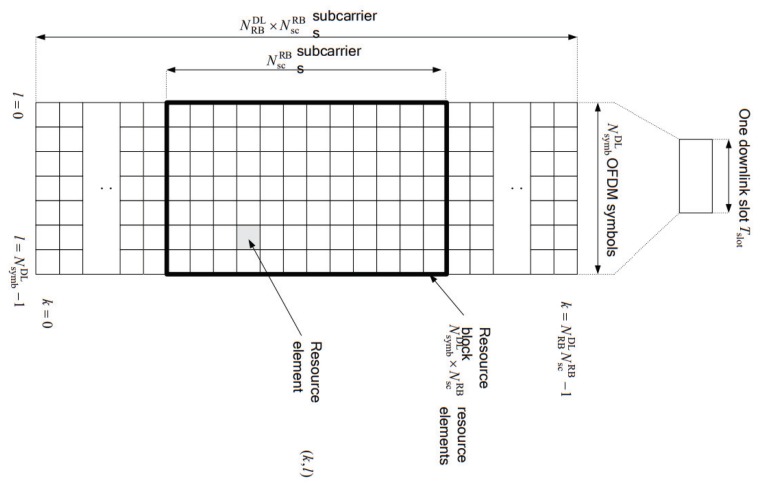
Downlink resource grid [21].

(1) *Waveform*: Although several candidate waveforms for uplink were proposed, Rel-15 recently decided to use OFDM-based waveforms with cyclic prefix for 5G NR downlink and uplink.
(2) *Bandwidth and sub-carrier spacing*: The size of resource blocks (RBs) is determined by the product of the number of sub-carriers on the frequency-axis and the number of symbols on the time-axis, as shown in Figure 1 [21]. Bandwidth is set to 5 MHz in this paper. The maximum number of available RBs is 25 at bandwidth of 5 MHz. When sub-carrier spacing is applied to 15 kHz, 12 sub-carriers are allocated in one RB on the frequency-axis and 14 symbols are allocated per sub-frame on the time-axis. A sub-frame consists of two slots and one slot consists of seven symbols. Assuming that the full band of 5 MHz is assigned to UE, 4200 resource elements (REs) can be allocated to UE for one sub-frame since 4200 REs are calculated by 25 RBs × 12 sub-carriers × 7 symbols × 2 slots per sub-frame. In other words, one RE is equivalent to the minimum resource for a symbol time on the time-axis and a sub-carrier on the frequency-axis.
(3) *Channel coding*: 5G NR replaces the previously used Turbo-code to LDPC coding. The number of the information bits and the number of the encoded bit are varied depending on channel coding schemes, the number of RBs, and the modulation scheme. TB size represents the number of information bits that can be transmitted throughout 4200 REs and it is denoted as *A*. After encoding TB of length *A* by channel coding scheme, the output is to be encoded bits of length *E*. Table 3 summarizes values of *A* and *E* according to the turbo code and LDPC when using 25 RBs per sub-frame. $M_{order}$ denotes modulation order and its values are 2, 4, and 6 for QPSK, 16QAM, and 64QAM, respectively.
(4) *Channel model*: Ideally, transmit signal is transferred over AWGN channel after LDPC encoding and rate matching. In practice, we experimented with fading channel environments in which CDL-A and TDL-A are utilized in SISO and 2 × 2 MIMO environments.

### 3.2. Preliminary Phase for 5G NR L2S Mapping

To perform L2S mapping, data of post-processing SINRs corresponding to allocated REs and BLERs is required. The procedure to extract raw data of post-processing SINRs and BLER from LLS is considered as a preliminary phase for L2S mapping. In AWGN environment, methods to extract raw data for L2S mapping are almost the same as the performance evaluation of stand-alone LLS. However, methods to extract raw data for L2S mapping in the fading channel environment are different from existing LLS evaluation methods. The preliminary phase for 5G NR L2S mapping is discussed in this subsection.

We consider fading channel models such as CDL or TDL specified in 3GPP TR 38.900 under downlink. As mentioned in Section 3.1, 4200 symbols are transmitted through 4200 REs for a sub-frame. Whenever one TB is generated every sub-frame, 4200 received SNRs corresponding 4200 REs can be measured on a UE side. Since a wide bandwidth of 5 MHz is used, the channel is varying on the frequency-axis domain but it does not change much on the time-axis. Therefore, we consider the received signal of 300 sub-carriers (i.e., 25 RBs × 12 sub-carriers × 1 symbol = 300 REs) at the first symbol-time to eliminate redundancy and reduce calculation complexity.

The received signal from the *k*th sub-carrier (1≤k≤300) at the first symbol-time in a sub-frame is expressed as follows:

(1) $$\begin{array}{c} y_{k}=H_{k}W_{k}s_{k}+n_{k}, \end{array}$$

where $y_{k}\in C^{N_{R}\times 1}$ is a received signal vector on the *k*th sub-carrier, $H_{k}\in C^{N_{R}\times N_{T}}$ is the MIMO channel matrix on the *k*th RE, $W_{k}\in C^{N_{T}\times N_{T}}$ is the precoder matrix on the *k*th sub-carrier, $s_{k}\in C^{N_{T}\times 1}$ is a transmitted symbol vector on the *k*th sub-carrier, and $n_{k}∼\mathrm{CN}\left(0,\sigma^{2}I_{N_{R}}\right)$ is a Gaussian noise vector on the *k*th sub-carrier. $N_{T}$ denotes the number of transmit antenna and $N_{R}$ denotes the number of receiver antennas.

A receiver filter on the *k*th sub-carrier by zero-forcing (ZF) and minimum mean square error (MMSE), $G_{k}$, is given as follows:

(2) $$\begin{array}{c} G_{k}^{}={\left(H_{k}^{H}H_{k}\right)}^{-1}H^{H},\;\;\;\mathrm{for}\mathrm{ZF},\\ G_{k}^{}={\left(H_{k}^{H}H_{k}+\sigma^{2}I_{N_{R}}\right)}^{-1}H_{k}^{H},\;\;\;\mathrm{for}\mathrm{MMSE}, \end{array}$$

where $G_{k}\in C^{N_{L}\times N_{R}}$ denotes a receiver filter matrix, and $N_{L}$ denotes the number of spatial transmission layers.

The estimated received symbol vector on the *k*th sub-carrier by a receiver filter, $x_{k}$, is expressed as follows:

(3) $$\begin{array}{c} x_{k}=G_{k}y_{k}=G_{k}H_{k}W_{k}s_{k}+G_{k}n_{k}, \end{array}$$

where $x_{k}\in C^{N_{L}\times 1}$.

Post-processing SINR of the *k*th sub-carrier at the *m*th layer is calculated as follows:

(4) $$SINR_{k}\left(m\right)=\frac{|{\left[G_{k}H_{k}W_{k}\right]}_{\left(m,m\right)}{|}^{2}}{\sum_{i=1,i\neq m}^{N_{L}}|{\left[G_{k}H_{k}W_{k}\right]}_{\left(m,i\right)}{|}^{2}+\sigma^{2}\sum_{i=1}^{N_{R}}{\left|{\left[G_{k}\right]}_{\left(m,i\right)}\right|}^{2}}$$

where ${\left[\cdot \right]}_{\left(m,i\right)}$ is a signal in row *m* and column *i* of matrix. The numerator term is the desired signal at the *m*th layer, whereas the first term of denominator is the sum of the inter-stream interference signals and the second term is the filtered noise.

Since each layer is independent and identically distributed, in the case of a large number of simulation, it does not matter to collect raw data from how many layers or which layer. For simplicity of experiment, raw data of post-processing SINRs and BLER is collected at the first layer. Accordingly, the variable *m* can be replaced to a constant 1 with the meaning of the first layer, and then it can be removed. Consequently, Equation (4) can be expressed to Equation (5) as follows:

(5) $$SINR_{k}=\frac{|{\left[G_{k}H_{k}W_{k}\right]}_{\left(1,1\right)}{|}^{2}}{\sum_{i=2}^{N_{L}}|{\left[G_{k}H_{k}W_{k}\right]}_{\left(1,i\right)}{|}^{2}+\sigma^{2}\sum_{i=1}^{N_{R}}{\left|{\left[G_{k}\right]}_{\left(1,i\right)}\right|}^{2}}$$

In a SISO case, *m*, $N_{T}$, $N_{R}$, and $N_{L}$ set to be 1, and there is no inter-stream interference. Thus, the post-processing SINR at the *k*th sub-carrier is expressed as follows:

(6) $$SINR_{k}=\frac{|H_{k}{|}^{2}}{\sigma^{2}}$$

For convenience of expression, post-processing SINRs ($SINR_{k}$) is denoted by $\gamma_{k}$.

One thousand TBs are repeatedly transmitted for a given *input SNR* under the same channel condition. CRC error checking is applied to determine whether the TB is successfully decoded. UE transmits *ACK* by successful TB decoding among transmitted TBs, whereas it transmits *NACK* by failed decoding. Therefore, BLER is calculated as follows:

(7) $$BLER=\frac{\mathrm{The}\mathrm{total}\mathrm{number}\mathrm{of}\mathrm{received}\mathrm{NACKs}}{\mathrm{The}\mathrm{total}\mathrm{number}\mathrm{of}\mathrm{transmitted}\mathrm{TBs}}\times 100\;\;\;\left[\%\right].$$

At the next *input SNR* under the same channel, 1000 TBs are transmitted recurrently. Correspondingly, the data of ($\gamma_{1}$, ⋯, $\gamma_{N_{RE}}$, *BLER*) is collected for a given *input SNR* and $N_{RE}$ REs where $N_{RE}$ is the number of allocated sub-carriers per symbol, i.e., 300. Thus, raw data of (*input SNR*, $\gamma_{1}$, ⋯, $\gamma_{N_{RE}}$, BLER) is gathered for L2S mapping. We describe LLS procedure to extract raw data as shown in Algorithm 1. Especially, the condition of channel must not be changed during the generation of 1000 TBs over all SNRs, while the condition of channel is changed at every simulation. The input SNR should be adjusted so that values of BLER are measured within the range of 0.01 and 0.9. When the value of measured BLER with 0 is used, $log_{10}\left(\mathrm{measured}\mathrm{BLER}\right)$ is to be negative infinite, as shown in Equation (9). BLER with 1 is meaningless data since all TBs failed. Since MSE is calculated from measured BLERs, MSE is dependent by measured BLERs. The exact value of L2S mapping can not be derived if the BLER value does not appear evenly in BLER range from 0.01 to 0.9. Therefore, equally distributed BLERs within the range from 0.01 to 0.9 should be measured to find the values of L2S mapping parameters.

### 3.3. Schematic of 5G NR L2S Mapping

**Figure 2:**
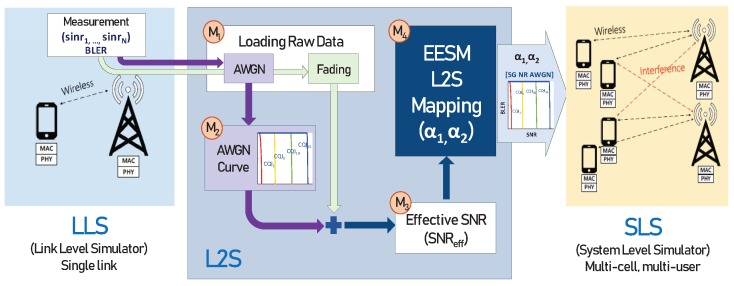
Schematic of 5G NR L2S mapping.

L2S mapping method has two aims to map various SNRs over allocated REs to one averaged SNR as well as to reduce system overload in SLS. The overall procedure of the L2S mapping is shown in Figure 2 where L2S mapping method basically receives raw data of $\gamma_{1},\cdots ,\gamma_{N_{RE}}$ and BLER from the LLS and then delivers two mapping parameters of ($\alpha_{1}$, $\alpha_{2}$) and 5G NR AWGN to the SLS. Our L2S mapping is composed of four modules: $M_{1}$, loading raw data; $M_{2}$, AWGN curve; $M_{3}$, Effective SNR (ESM); and $M_{4}$, exponential ESM (EESM) L2S mapping. The operation and function of each module are explained below.

(1) *Module $M_{1}$: Loading raw data*
  $M_{1}$ module reads stored data on the preliminary phase in Section 3.2. The format of raw data is composed of *x* rows and *y* columns for each CQI. The value of *x* is the number of input SNRs× the number of simulations (Sim) and the value of *y* is $N_{RE}$+2. As mentioned in Section 3.1, the number of allocated sub-carriers per sub-frame is $N_{RE}$. The first column is a given *input SNR*, the last column is BLER, and the rest of columns mean $\gamma_{1}$, ⋯, $\gamma_{N_{RE}}$. The data loading is performed for AWGN channel and all fading channels, respectively.
(2) *Module $M_{2}$: AWGN curve*
  $M_{2}$ module only applies to AWGN channel. It gets AWGN raw data from $M_{1}$ module and makes AWGN fitting curve for SNR versus BLER in the range of all CQIs. The AWGN fitting curve is generated from the relation of ($\gamma_{1}$, ⋯, $\gamma_{N_{RE}}$) and BLER. Generally, the fitting curve is induced from an exponential function or regression curve of machine learning.
(3) *Module* $M_{3}$ *: Effective SNR*
  $M_{3}$ module calculates an effective SNR applying $\alpha_{1}$ and $\alpha_{2}$ when a UE measures $\gamma_{1},\cdots ,\gamma_{N_{RE}}$, and it is expressed as follows [5,6]:
  (8) $$\gamma_{\mathrm{eff}}^{}\left(\alpha_{1},\alpha_{2}\right)=-\alpha_{1}\ln\left(\frac{1}{N}\sum_{k=1}^{N}\exp(-\frac{\gamma_{k}^{}}{\alpha_{2}})\right),$$
  where $\alpha_{1}$ and $\alpha_{2}$ are determined after optimization in $M_{4}$.
  In fact, SLS only measures $\left\{\gamma_{1},\gamma_{2},\cdots ,\gamma_{N_{NE}}\right\}$ without decoding. To estimate error for a received TB, SLS calculates an effective SNR from Equation (8) using $\alpha_{1}$ and $\alpha_{2}$. The values of $\alpha_{1}$ and $\alpha_{2}$ are already reported to SLS through a L2S mapping method.
(4) *Module $M_{4}$: EESM based L2S Mapping*
  $M_{4}$ module finds optimal mapping parameters of $\alpha_{1}$ and $\alpha_{2}$ for a given CQI and a channel type. After performing *M* snapshots, we calculate mean square error (MSE) as follows [5,6]:
  (9) $$\mathrm{MSE}\left(\alpha_{1},\alpha_{2}\right)=\sum_{i=1}^{M}{\left[\log_{10}BLER_{i}-\log_{10}BLER_{R}\left(\gamma_{\mathrm{eff}}^{i}\left(\alpha_{1},\alpha_{2}\right)\right)\right]}^{2}.$$
  where *M* denotes the total number of simulated snapshots and $BLER_{i}$ denotes the BLER measured from the *i*th post-processing SINR values. In addition, $\gamma_{\mathrm{eff}}^{i}\left(\alpha_{1},\alpha_{2}\right)$ is calculated by Equation (8) at the *i*th snapshot, and $BLER_{R}$ is the value of BLER on AWGN channel. Furthermore, $BLER_{R}\left(x\right)$ denotes output BLER corresponding to input *x* from AWGN fitting curve. (It is plotted in module $M_{2}$)
  To find optimal $\alpha_{1}^{*}$ and $\alpha_{2}^{*}$, we minimize MSE for the entire range of ($\alpha_{1}$, $\alpha_{2}$) as follows:
  (10) $$\begin{array}{c} \left(\alpha_{1}^{*},\alpha_{2}^{*}\right)=\underset{\left(\alpha_{1},\alpha_{2}\right)}{arg min}\;\;\;\;\mathrm{MSE}\left(\alpha_{1},\alpha_{2}\right). \end{array}$$
  Since the BLERs vs. SNR varies depending on the modulation method, code block size and code rate, we should find $\alpha_{1}^{*}$ and $\alpha_{2}^{*}$ for a given condition.

**Algorithm 1:** Extract raw data.

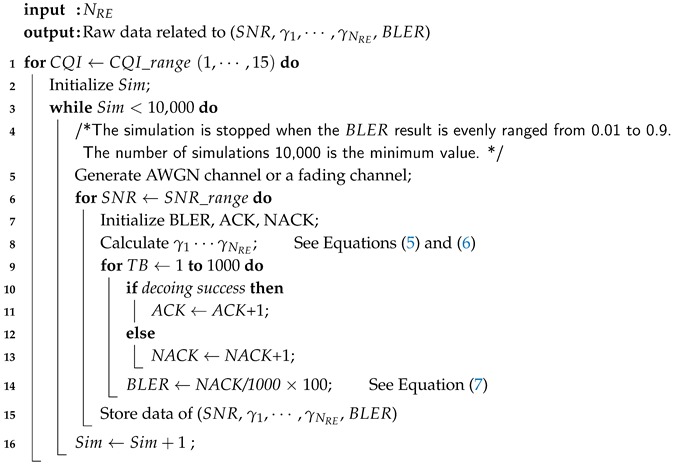



## 4. Machine Learning Based Effective SNR Mapping Procedure

**Figure 3:**
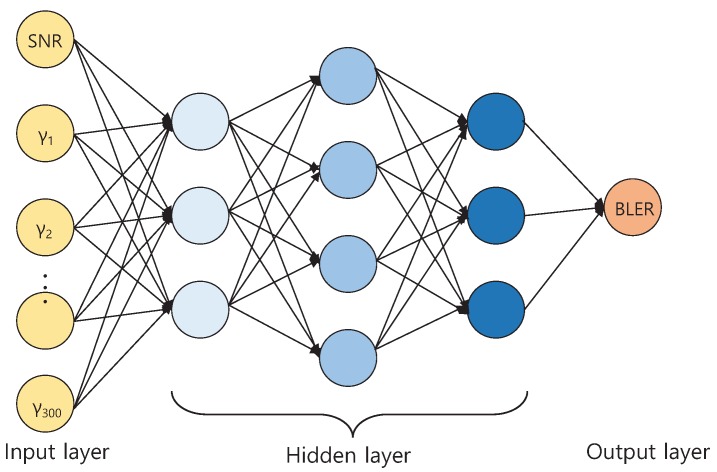
Deep neural network.

The number of simulations was 10,000 for each input SNR and each CQI and 1000 TBs were generated for each simulation. BLER was determined for transmitted 1000 TBs. Thus, the size of raw data of (SNR, $\gamma_{1},\cdots ,\gamma_{N_{RE}}$, BLER) to collect from LLS, i.e., the total number of simulation results per CQI was the product of the number of SNR values and the the number of simulations. In general, since the size of raw data from the LLS is large, obtaining the optimal parameters of $\alpha_{1}^{*}$ and $\alpha_{2}^{*}$ through heuristic search with Equation (10) is burdensome. Thus, it was necessary to reduce the computation complexity as well as to improve the accuracy. As shown in this section, we used Python Tensorflow framework and applied the DNN regression method to make an AWGN fitting curve of module $M_{2}$ in Section 3.3. The algorithm of DNN regression is described in Algorithm 2 and Figure 3.

(1) We utilized the DNN regression method instead of the best fitting curve to obtain the BLER curve in AWGN channels. The DNN consists of several hidden layers between the input and output layers. Hidden layers of (100, 200, 100) layers were used in Adagrad optimizer [22]. As the number of hidden layers and the number of nodes at each hidden layer increased, MSE decreased. However, the improvement of MSE was saturated at three hidden layers with the number of nodes of (100, 200, 100), as shown in Figure 3. Learning rate was set to 0.1, which implies how quickly it is tuned to the target SNR value. The regularization strength to prevent over-fitting was set to 0.001. The sigmoid function $1/(1+e^{-x})$ was used as an activation function in hidden layers.
(2) DNN regression continued training for SNRs and BLERs on AWGN channel with the learning rate at each epoch. The number of training data was 4000.
(3) After training data, BLERs were predicted for test set of SNRs. Finally, we obtained an enhanced AWGN curve of SNRs and BLERs.

**Algorithm 2:** DNN regression. /* Configure DNN regression                          */
**1** regressor = learn.DNNRegressor(feature_columns, hidden_units = [100, 200, 100], optimizer =  tf.train.ProximalAdagradOptimizer( learning_rate = 0.1, l1_regularization_strength = 0.001),  activation_fn = tf.nn.sigmoid) /* Train measured data up to 4000 times                    */
**2** input_training_fn ← (awgn_snr, awgn_bler) **3** regressor.fit(input_fn = input_training_fn, steps = 4000) /* Predict of BLERs for test SNRs                       */
**4** input_reff_fn ← snr range
**5** predictions = list(regressor.predict_scores(input_fn = input_reff_fn))
**6** regressed_bler = np.asarray(predictions)


Next, we applied the optimization algorithm to efficiently find $\left(\alpha_{1}^{*},\alpha_{2}^{*}\right)$, which is summarized in Algorithm 3.

(1) To find the optimal parameters $\left(\alpha_{1}^{*},\alpha_{2}^{*}\right)$, we loaded raw data from module $M_{1}$ in Section 3.2.
(2) In the ML scheme, the loss function is defined as the difference between the calculated effective SNR value from Algorithm 3 and AWGN SNR obtained from Algorithm 2 at the same BLER. We calculated loss as the expectation of loss function over BLERs. The loss function and MSE of Equation (9) were used almost synonymously.
(3) We applied optimization algorithms, Adagrad and RMSProp, to find the optimal parameters that minimize the loss function. Since Adagrad and RMSProp adapt the learning rate to the parameters, they eliminate the need to manually tune the learning rate. Adagrad optimizer adapts the learning rates by scaling them inversely proportional to the sum of the historical squared values of the gradient. In contrast, RMSprop optimizer modifies AdaGrad for a nonconvex setting by changing gradient accumulation into exponentially weighted moving average [23].
(4) With the optimal parameters, the mean squared error (MSE) was calculated by
  (11) $$\mathrm{MSE}=\sum_{i=1}^{M}{\left[\log_{10}BLER_{i}-\log_{10}BLER_{R}\left(\gamma_{\mathrm{eff}}^{i}\left(\alpha_{1}^{*},\alpha_{2}^{*}\right)\right)\right]}^{2}.$$

**Algorithm 3:** Find optimal $\alpha_{1}$ and $\alpha_{2}$. /* Load data on Fading channel  ;                          */
**1** snr_k ← post-processing SINRs, bler ← BLER
 /* Calculate $\gamma_{\mathrm{eff}}$ with $\alpha_{1}$ and $\alpha_{2}$                            */
**2** snr_eff = -1 * alpha1 * tf.log(tf.reduce_mean (tf.exp(-1*snr_k/alpha2), axis = 1))
 /* Decide target SNR by regression                           */
**3** target_snr ← predicted snr corresponding to BLER
 /* Calculate loss function                               */
**4** loss = tf.reduce_sum(tf.abs(tf.subtract (target_snr,snr_eff)))
 /* Select a training algorithm between Adagrad and RMSprop; Adagrad is selected in this case.                                 */
**5** train = tf.train.AdagradOptimizer(0.1).minimize(loss) /* train = tf.train.RMSPropOptimizer(0.1).minimize(loss) ← when RMSProp is 
 selected.                                        */
 /* Training data 4000 times                              */
**6** with tf.Session() as sess:
**7** sess.run(init)
**8** for i in range(4000):
**9**  sess.run(train)
 /* Calculate MSE in test data set                           */
**10** regressed_bler ← estimated BLER, y_data ← BLER
**11** mse = np.mean(np.square(np.subtract(np.asarray(y_data), np.asarray(regressed_bler))))

## 5. Numerical Results and Analysis

**Figure 4:**
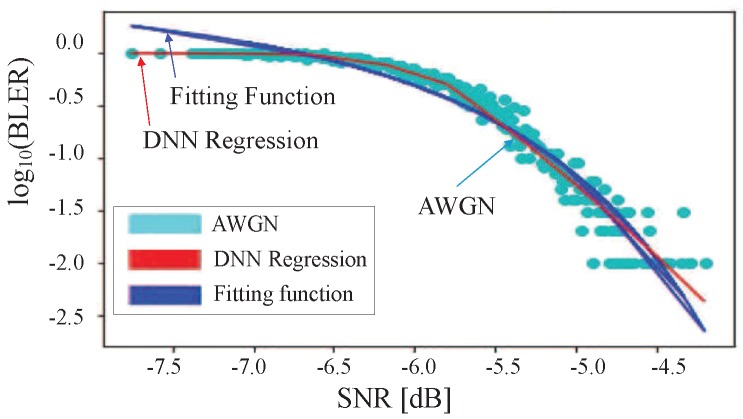
SNR vs. BLER for CQI 1 under 4G AWGN channel [12].

Table 2 shows the system parameters used. To make AWGN curve, we used a DNN regression method. Actually, we already showed that a DNN regression is a more suitable method than a fitting function under 4G LTE-A systems in our previous work [12]. The fitting curve became over-fitted as BLER approached zero, as shown in Figure 4. In contrast, a DNN regression followed AWGN curve as closely as possible through a multi-dimensional mapping. Moreover, Table 4 shows MSE results for fitting function (“FIT”) and DNN regression (“DNN”) for all CQIs under 4G LTE-A systems. Actually, the deep neural network gave little improvement compared to fitting algorithm under AWGN channel condition since AWGN was an almost static channel environment. Accordingly, in this study, 5G NR AWGN curve was generated with a DNN regression method without comparison of FIT and DNN.

### 5.1. AWGN Curve

**Figure 5:**
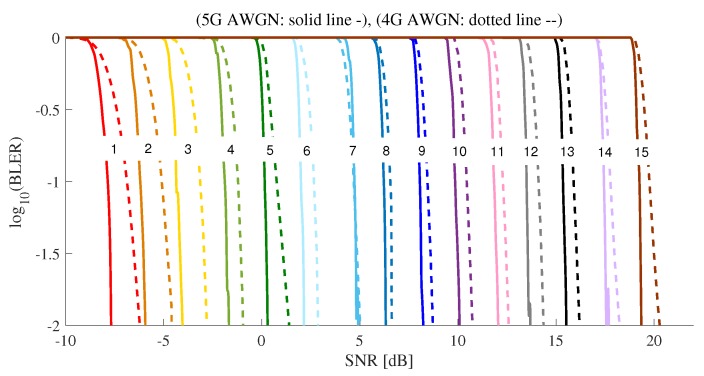
The BLER comparison between 4G AWGN and 5G AWGN.

Figure 5 shows BLER performance for 4G AWGN and 5G AWGN. 4G LTE-A systems and 5G NR systems use Turbo-code and LDPC as channel coding schemes, respectively. The size of TB and their coding rates varied slightly according to channel coding schemes, as shown in Table 3. After applying the channel coding shown in Table 3, the performances of 4G AWGN and 5G NR AWGN were evaluated. 5G NR AWGN outperformed 4G AWGN, especially better for small CQL values.

3GPP 4G LTE and 5G NR-standards are specified to set the target BLER of 0.1, i.e., 10%. As SNR increased, BLER decreased. SNR could be measured immediately on SLS, but the calculation of BLER was burdensome because CRC decoding had to be performed. When measured BLER is lower than target BLER, a UE can choose the index of high CQI to support high data rate. Under an unknown BLER, we found the value of SNR satisfying the target BLER of 0.1 through the simulation results and induced Equation (12) for CQI and SNR.

**Figure 6:**
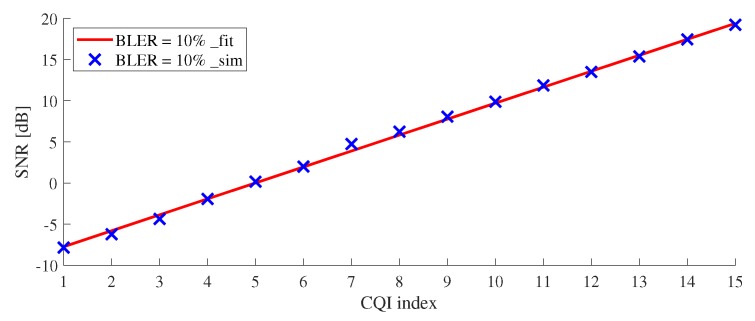
SNR threshold for all CQIs under 5G AWGN.

From the AWGN curve shown in Figure 4, SINR threshold at BLER = 0.1 (10%) can be derived, as shown in Table 5. In practical 4G/5G systems, if an estimated BLER exceeds 0.1, TB transmission is considered as failure. Otherwise, TB transmission is considered as success. We plot SINR threshold at BLER = 10% for the range of all CQIs in Figure 6. From this plot, we derive a linear equation as follows:

(12) $$SNR_{th}\left(i\right)=1.938\times i-9.682\;\;\left[dB\right],i=1,\cdots ,15,$$

where *i* means CQI index. $SNR_{th}\left(i\right)$ is SINR threshold for CQI index *i* at 10% BLER.

### 5.2. Optimal Values of $\alpha_{1}$ and $\alpha_{2}$

To find optimal values of $\alpha_{1}$ and $\alpha_{2}$, two optimizers, Adgrad and RMSProp, were used. Table 6 shows optimal parameters $\left(\alpha_{1}^{*},\alpha_{2}^{*}\right)$ in the case of 5G CDL-A SISO, whereas Table 7 shows optimal parameters $\left(\alpha_{1}^{*},\alpha_{2}^{*}\right)$ in the case of 5G TDL-A 2 × 2 MIMO. The MSE performances of both optimizers were similar. In our previous work [12], we analyzed the optimal EESM parameters in 4G LTE-A system. At that time, the MSE performance of RMSProp was better than that of AdaGrad. However, since 5G NR AWGN curve aws too steep, there was little difference in performance of the two optimizers. In the case of 2 × 2 MIMO, the variance of $sinr_{1}\cdots sinr_{N_{RE}}$ was larger than that of SISO, thus the MSE of MIMO was larger than MSE of SISO.

### 5.3. Simulation Validation

**Figure 7:**
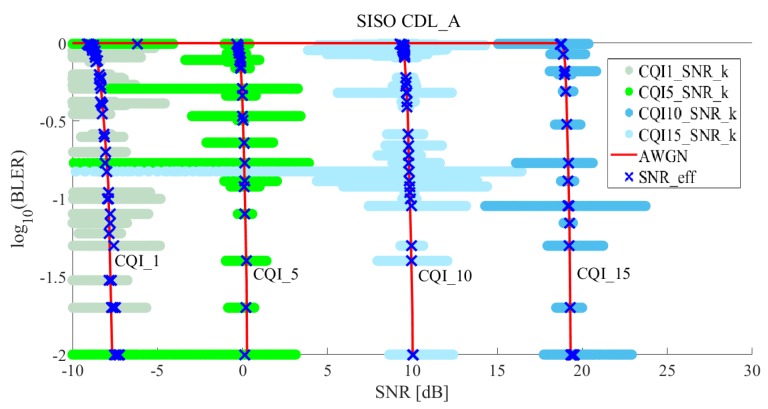
Effective SNR mapping results of the proposed ML-based EESM method under 5G CDL-A SISO (for CQI1, CQI5, CQI10, and CQI15).

**Figure 8:**
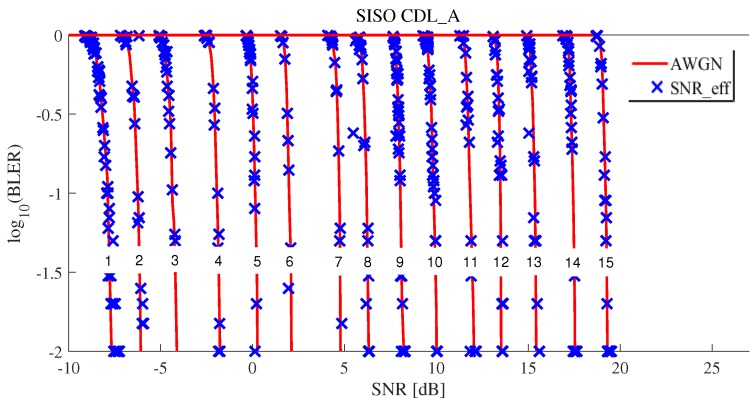
Effective SNR mapping results of the proposed ML-based EESM method under 5G CDL-A SISO for all CQIs.

Figure 7 and Figure 8 show the results of effective SNR mapping by the proposed ML-based EESM method in the case of 5G CDL-A SISO. “CQI1_SNR_k”, “CQI5_SNR_k”, “CQI10_SNR_k”, and “CQI15_SNR_k” indicate the post-processing SINRs $sinr_{k}$, as mentioned in Equation (6). Since the number of allocated REs $N_{RE}$ was 300 (25 RB × 12 sub-carriers × 1 symbol time = 300 REs), $sinr_{1}\cdots sinr_{N_{RE}}$ corresponding to BLER are plotted in Figure 7. When wide-band signal passed through a fading channel, each RE suffered different fading. Thus, the results show that $N_{RE}$ $sinr_{k}$s spread widely. AWGN indicates SNR and BLERs at CQI 1, 5, 10 and 15 in 5G NR AWGN, as shown in Figure 5. To perform EESM L2S mapping, we applied optimal parameters of $\alpha_{1}^{*}$ and $\alpha_{2}^{*}$ obtained using RMSProp optimizer, as shown in Table 6. Blue x mark presents effective SNRs and the results show that widespread $sinr_{1}\cdots sinr_{N_{RE}}$ were mapped to one effective SNR on the AWGN curve. Figure 8 shows how well is the effective SNR mapped to the AWGN for all CQIs. The results show that most blue x marks were on the AWGN line. With these results, we observed that the proposed method predicted the BLER quite well.

**Figure 9:**
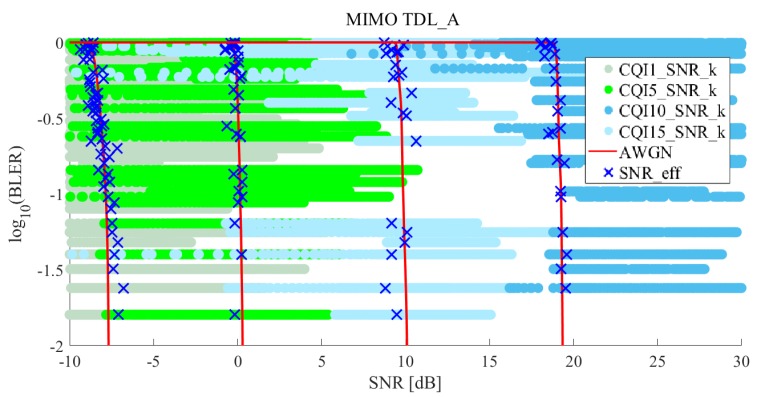
Effective SNR mapping results of the proposed ML-based EESM method under 5G TDL-A 2 × 2 MIMO (for CQI1, CQI5, CQI10, and CQI15).

**Figure 10:**
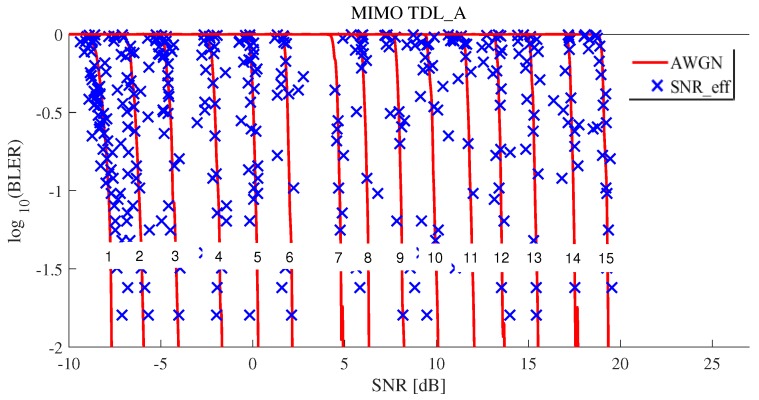
Effective SNR mapping results of the proposed ML-based EESM method under 5G TDL-A 2 × 2 MIMO for all CQIs.

Figure 9 and Figure 10 show the results of effective SNR mapping by the proposed ML-based EESM method in the case of 5G TDL-A 2 × 2 MIMO. MIMO cases were also similar to SISO cases from the analytical point of view. As shown in Figure 9, $sinr_{1}\cdots sinr_{N_{RE}}$ were more widespread compared to SISO case shown in Figure 9. Therefore, since the variance of $sinr_{1}\cdots sinr_{N_{RE}}$ was larger than that of SISO, MSE of MIMO was also larger than that of SISO. Figure 10 shows how well is the effective SNR mapped to the AWGN for all CQIs under 2×2 MIMO. Compared to SISO results, there were a few blue x that were slightly off the AWGN line.

## 6. Conclusions

We proposed a novel link-to-system (L2S) mapping method based on machine learning (ML) for 5G new radio (NR) simulators, which reduces the computational complexity as well as improves the prediction accuracy of block error rates (BLERs) prediction. The performance of the proposed ML-based L2S mapping technique was validated by utilizing link-level simulator (LLS) of the 5G K-simulator. In particular, we adopted the optimizers such Adagrad and RMSProp for obtaining parameters for effective exponential signal-to-noise ratio mapping (EESM).

As a further study, we will investigate the ML-based decision process for the system-level simulator (SLS) to determine whether a received frame is successfully decoded without the L2S mapping procedure.

## Figures and Tables

**Table 1:** Abbreviations.

Abbreviation: Full Name	Abbreviation: Full Name
AWGN: additive white Gaussian noise	BLER: block error rate
CQI: channel quality indication	CDL: clustered delay line
DNN: deep neural network	EESM: exponential effective SNR mapping
eNB: evolved Node-B	LDPC: Low-density parity-check
LTE-A: long term evolution-advanced	LLS: link-level simulator
L2S: link-to-system	MIMO: multiple input multiple output
ML: machine learning	MMSE: minimum mean square error
MSE: mean squared error	NS: network-level simulator
NR: new radio	RB: resource block
RE: resource element	SINR: signal-to-interference plus noise ratio
SISO: single input single output	SLS: system-level simulator
SNR: signal-to-noise ratio	TB: transport block
TDL: tapped delay line	UE: user equipment

**Table 2:** System configuration/setup.

Parameters	Values
Waveform	OFDM
Carrier frequency	2.8 GHz
Bandwidth	5 MHz
Sub-carrier spacing	Δf=15 kHz
Channel Estimation	Perfect, MMSE
The number of allocated RBs to a UE	25 RBs
The number of sub-carriers per RB	12 sub-carriers
The number of symbols per slot	7 symbols
The number of slots per sub-frame	2 slots
Channel Coding	Turbo-Code (4G), LDPC (5G)
Channel Model	AWGN, CDL-A (SISO), TDL-A (2×2 MIMO)

**Table 3:** Channel Quality Indication (CQI) table for turbo-code and LDPC.

		LDPC				Turbo Code			
CQI	Mod.	A	E	$C_{R}$	η	A	E	$C_{R}$	η
		***[Bits]***	***[Bits]***	A/E	$C_{R}$×$M_{\mathrm{order}}$	***[Bits]***	***[Bits]***	A/E	$C_{R}$×$M_{\mathrm{order}}$
1	QPSK	584	8000	0.0762	0.1523	608	7800	0.0779	0.1559
2	QPSK	912	8000	0.1172	0.2344	928	7800	0.1190	0.2379
3	QPSK	1480	8000	0.1885	0.3770	1480	7800	0.1897	0.3795
4	QPSK	2384	8000	0.3008	0.6016	2408	7800	0.3087	0.6174
5	QPSK	3480	8000	0.4385	0.8770	3496	7800	0.4482	0.8964
6	QPSK	4680	8000	0.5879	1.1758	4608	7800	0.5908	1.1815
7	16QAM	5880	16,000	0.3691	1.4766	5760	15,600	0.3692	1.4769
8	16QAM	7632	16,000	0.4785	1.9141	7424	15,600	0.4759	1.9036
9	16QAM	9600	16,000	0.6016	2.4063	9480	15,600	0.6077	2.4308
10	64QAM	10,896	24,000	0.4551	2.7305	10,760	23,400	0.4598	2.7590
11	64QAM	13,264	24,000	0.5537	3.3223	13,064	23,400	0.5583	3.3497
12	64QAM	15,584	24,000	0.6504	3.9023	15,112	23,400	0.6458	3.8749
13	64QAM	18,072	24,000	0.7539	4.5234	17,424	23,400	0.7446	4.4677
14	64QAM	20,440	24,000	0.8525	5.1152	19,968	23,400	0.8533	5.1200
15	64QAM	22,192	24,000	0.9258	5.5547	21,504	23,400	0.9190	5.5138

**Table 4:** The comparison of MSE under 4G AWGN channel [12].

CQI Index	CQI 1	CQI 2	CQI 3	CQI 4	CQI 5	CQI 6	CQI 7	CQI 8
FIT	0.033	0.027	0.033	0.038	0.017	0.032	0.017	0.012
DNN	0.018	0.014	0.016	0.013	0.014	0.011	0.015	0.016
CQI Index	CQI 9	CQI 10	CQI 11	CQI 12	CQI 13	CQI 14	CQI 15	-
FIT	0.019	0.021	0.011	0.025	0.013	0.015	0.030	-
DNN	0.011	0.008	0.013	0.013	0.010	0.013	0.010	-

**Table 5:** The values of SNR Threshold satisfying 10% BLER.

CQI Index	CQI 1	CQI 2	CQI 3	CQI 4	CQI 5	CQI 6	CQI 7	CQI 8
SNR Threshold [dB]	−7.8474	−6.2369	−4.3591	−1.9319	0.1509	1.9976	4.7278	6.2231
CQI Index	CQI 9	CQI 10	CQI 11	CQI 12	CQI 13	CQI 14	CQI 15	-
SNR Threshold [dB]	8.0591	9.8585	11.8432	13.4893	15.3598	17.4435	19.2155	-

**Table 6:** Optimal parameters $\left(\alpha_{1}^{*},\alpha_{2}^{*}\right)$ in 5G CDL-A SISO.

CQI	AdaGrad				RMSProp		
	$\alpha_{1}$	$\alpha_{2}$	MSE		$\alpha_{1}$	$\alpha_{2}$	MSE
1	3.294	3.230	0.150		2.752	2.698	0.149
2	1.874	1.880	0.357		2.163	2.170	0.364
3	1.607	1.594	0.065		2.002	1.988	0.061
4	1.184	1.175	0.159		1.162	1.154	0.156
5	1.286	1.283	0.140		1.552	1.546	0.206
6	1.359	1.359	0.055		1.360	0.360	0.056
7	3.642	3.628	0.170		3.643	3.629	0.170
8	3.256	3.228	0.171		3.937	3.911	0.155
9	5.563	5.543	0.110		5.636	5.616	0.110
10	16.259	16.204	0.075		16.262	16.208	0.075
11	13.685	13.604	0.329		13.382	13.301	0.268
12	17.988	18.079	0.778		17.547	17.632	0.724
13	23.971	23.970	0.555		24.112	24.111	0.558
14	29.306	29.205	0.210		29.306	29.204	0.210
15	33.590	33.833	0.533		28.733	28.739	0.823

**Table 7:** Optimal parameters $\left(\alpha_{1}^{*},\alpha_{2}^{*}\right)$ in 5G TDL-A 2 × 2 MIMO.

CQI	AdaGrad				RMSProp		
	$\alpha_{1}$	$\alpha_{2}$	MSE		$\alpha_{1}$	$\alpha_{2}$	MSE
1	0.088	0.126	1.581		0.079	0.106	1.465
2	0.114	0.154	0.834		0.114	0.152	0.969
3	0.200	0.252	0.730		0.208	0.260	1.070
4	0.280	0.306	0.867		0.301	0.339	0.976
5	0.511	0.525	0.859		0.513	0.528	0.865
6	0.718	0.729	2.072		0.718	0.729	2.070
7	2.664	3.554	1.526		2.664	3.553	1.528
8	2.465	2.665	1.538		2.465	2.664	1.525
9	3.200	4.695	1.782		3.203	4.700	1.784
10	6.532	8.174	1.824		6.338	7.900	1.777
11	6.588	8.619	0.703		5.969	7.634	0.688
12	9.985	11.538	1.125		10.021	11.661	1.135
13	11.190	14.225	1.393		11.19	14.224	1.399
14	15.933	19.071	0.973		19.562	23.828	2.784
15	20.410	38.530	1.399		19.062	34.963	1.099
